# NF-κB Protects NKT Cells from Tumor Necrosis Factor Receptor 1-induced Death

**DOI:** 10.1038/s41598-017-15461-y

**Published:** 2017-11-15

**Authors:** Amrendra Kumar, Laura E. Gordy, Jelena S. Bezbradica, Aleksandar K. Stanic, Timothy M. Hill, Mark R. Boothby, Luc Van Kaer, Sebastian Joyce

**Affiliations:** 1 Veterans Administration Tennessee Valley Healthcare System, Nashville, USA; 2 Department of Pathology, Microbiology & Immunology, Vanderbilt University School of Medicine, Nashville, Tennessee USA; 3 The Kennedy Institute of Rheumatology, University of Oxford, Oxford, UK; 4 Department of Obstetrics and Gynecology, University of Wisconsin-Madison, Madison, Wisconsin USA; 5 Department of Chemistry and Life Science, United States Military Academy, West Point, NY 10996 USA

## Abstract

Semi-invariant natural killer T (NKT) cells are innate-like lymphocytes with immunoregulatory properties. NKT cell survival during development requires signal processing by activated RelA/NF-κB. Nonetheless, the upstream signal(s) integrated by NF-κB in developing NKT cells remains incompletely defined. We show that the introgression of Bcl-x_L_-coding *Bcl2l1* transgene into NF-κB signalling-deficient *I*κ*BΔN* transgenic mouse rescues NKT cell development and differentiation in this mouse model. We reasoned that NF-κB activation was protecting developing NKT cells from death signals emanating either from high affinity agonist recognition by the T cell receptor (TCR) or from a death receptor, such as tumor necrosis factor receptor 1 (TNFR1) or Fas. Surprisingly, the single and combined deficiency in PKC-θ or CARMA-1—the two signal transducers at the NKT TCR proximal signalling node—only partially recapitulated the NKT cell deficiency observed in *IκBΔN* ^*tg*^ mouse. Accordingly, introgression of the *Bcl2l1* transgene into PKC-θ null mouse failed to rescue NKT cell development. Instead, TNFR1-deficiency, but not the Fas-deficiency, rescued NKT cell development in *IκBΔN* ^*tg*^ mice. Consistent with this finding, treatment of thymocytes with an antagonist of the inhibitor of κB kinase —which blocks downstream NF-κB activation— sensitized NKT cells to TNF-α-induced cell death *in vitro*. Hence, we conclude that signal integration by NF-κB protects developing NKT cells from death signals emanating from TNFR1, but not from the NKT TCR or Fas.

## Introduction

Natural killer T (NKT) cells are thymus-derived, innate-like lymphocytes that resemble both natural killer and T cells in phenotype and function. Based on the T cell receptor (TCR) α-chain expressed, NKT cells are broadly divided into two types: Type I NKT cells, the majority group, expresses an invariant TCR α-chain encoded by a conserved rearrangement between mouse *V*α14 and *J*α18 gene segments. This invariant α-chain predominantly pairs with mouse Vβ8.2 (and with Vβ7, 6, 2, and 14) TCR β-chain to express a semi-invariant NKT TCR. Type II NKT cells, the minority group, expresses more diverse TCR α- and β-chains. This report focuses on the semi-invariant NKT (hereinafter NKT) cells. In this regard, it is noteworthy that mouse, rat, human, and other mammalian species also develop functional NKT cells that express the orthologous semi-invariant TCR. Hence, findings in mice will have implications for understanding NKT cell development and function in other mammalian species including humans.

NKT cells control immune responses to microbial, allograft and self antigens. NKT cell functions are controlled by lipid ligand-presenting CD1d molecules. Consequently, NKT cells are capable of sensing cellular lipid homeostasis to maintain cardiovascular and metabolic health^1^. These seemingly divergent functions are mediated by the ability of NKT cells to rapidly produce pro-inflammatory and immunoregulatory cytokines, which permits these cells to interact with and transactivate cells of the innate and adaptive immune systems^2^. These properties of NKT cells are acquired during thymic development. Hence, insights into the mechanisms by which NKT cells develop, attain functional competence and are maintained can help understand the role these cells play in health and disease.

The unique phenotypic and functional features of NKT cells —especially their capacity to secrete cytokines promptly after thymic emigration^3^ —suggested that these cells might follow a distinct ontogenetic path. Commitment to the NKT cell lineage occurs at the CD4- and CD8-positive (DP) thymocyte stage^4–7^ whence self-lipid(s) bound CD1d/NKT TCR^8–11^ and homotypic/heterotypic SLAM-SLAM^12,13^ interactions lead to downstream PKCθ-NFκB^14^, NFAT-Egr2^15–17^, and SAP-Fyn^12,13,18^ activation, which turns-on a unique transcriptional programming mediated by the *Zbtb16*-encoded master transcription factor PLZF (pro-myelocytic leukaemia zinc finger). PLZF controls multiple molecular events distinguishing NKT cells from conventional T cells^19,20^. NK1.1-negative NKT cell precursors undergo several rounds of cell division^21,22^, upon which some remain within the thymus whereas the majority emigrate and populate the peripheral lymphoid organs^23^. Both the thymic and peripheral NK1.1-negative NKT cells undergo further maturation and become NK1.1-positive NKT cells. During this maturation process, NKT cells differentiate into subsets and acquire distinct effector behaviours^24–29,23^.

Several research groups^30–36^, including ours^14,37^, have reported that signal integration by NF-κB was essential for NKT cell development, survival, and function. RelA and RelB were shown to confer cell autonomous and cell extrinsic activities of NF-κB, respectively^30–32^. The cell-extrinsic NF-κB activity was required for IL-15 induction^32^, a cytokine essential for survival as well as for terminal NK1.1-negative to NK1.1^+^ NKT cell differentiation and effector maturation^38–47^. Likewise, the cell-intrinsic NF-κB activity was essential for the survival of NK1.1-negative NKT cells, as introgression of *Bcl2l1* transgene under the control of the *Lck* proximal promotor, which thereby encodes Bcl-x_L_ in thymocytes, rescued NKT cell numbers but not function^14^. Despite this understanding of the role of NF-κB in NKT cell development, a few key questions have remained unanswered: (*a*) Is signal integration by NF-κB essential for the survival of both immature NK1.1-negative and mature NK1.1^+^ NKT cells?; (*b*) Does NF-κB activation protect against TCR-induced NKT cell death?; and (*c*) What other death receptor(s) trigger NKT cell death during ontogeny? In seeking answers to these questions, we report herein that signal integration by NF-κB is essential for the survival of both immature NK1.1-negative and mature NK1.1^+^ NKT cells and to protect these cells from death signals induced by tumor necrosis factor (TNF)-α and emanating from TNF receptor superfamily 1a (TNFR1), but not the NKT TCR or Fas.

## Results

### Bcl-x_L_ overexpression rescues immature and mature NKT cell development in NF-κB signalling deficient mice

**Figure 1:**
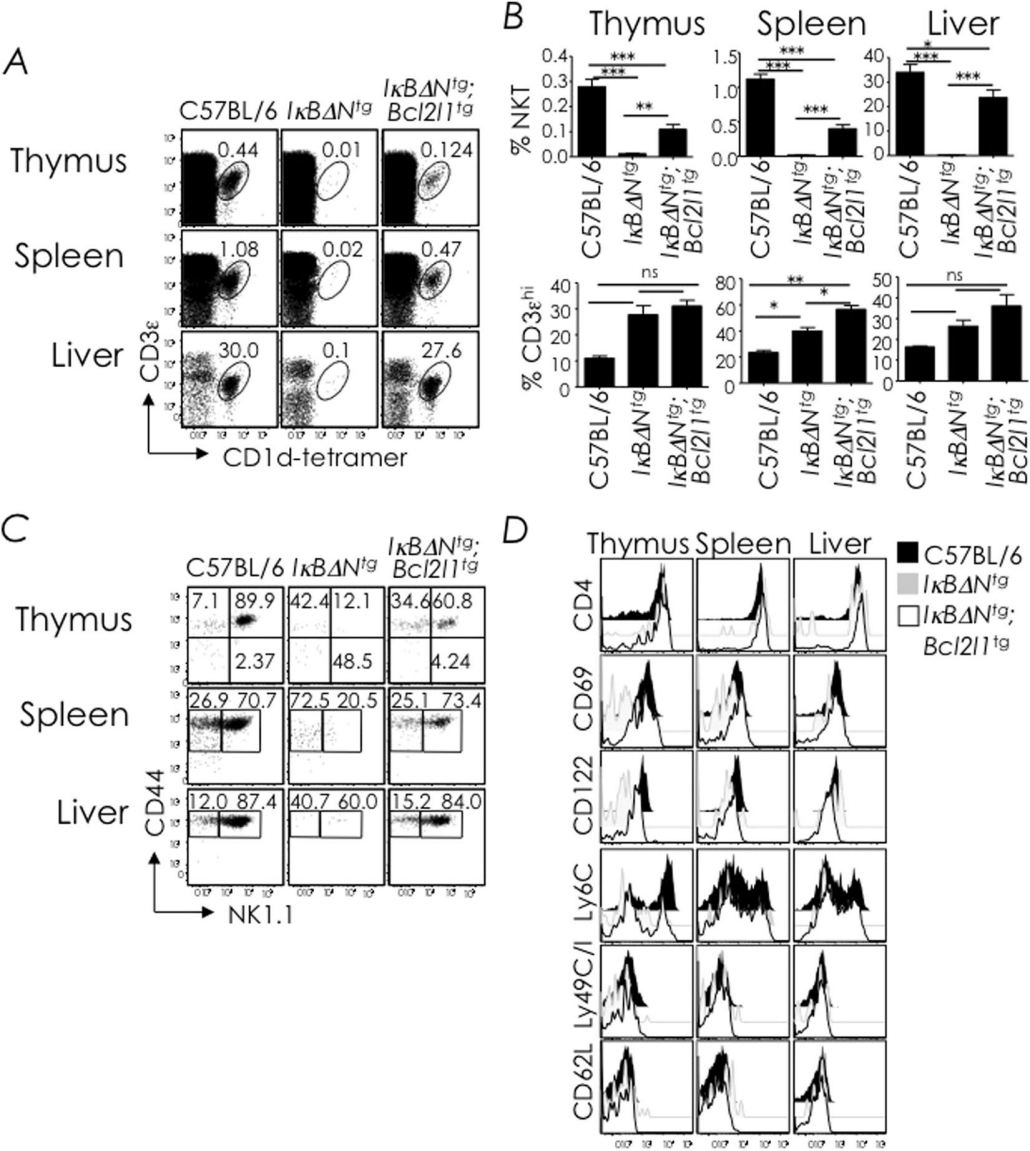
Bcl-x_L_ overexpression rescues NKT cell development in NFκB-signalling deficient mice. (**A**) Thymic, splenic and hepatic NKT cells from C57BL/6 (*n* = 4), B6-*I*κ*B*α*ΔN* ^*tg*^ (*n* = 8) and B6-*I*κ*B*α*ΔN* ^*tg*^;*Bcl2l1* ^tg^ (*n* = 4) mice were identified as CD3ε^+^tetramer^+^ cells within electronically gated CD8^lo^ thymocytes or B220^lo^ splenocytes. Numbers are % NKT cells among total leukocytes within each organ. (**B**) % of NKT cells (top) and CD3ε^hi^ cells in thymus spleen and liver, Data are mean ± standard error (sem) from 3 independent experiments. (**C**) NKT cell developmental stages were identified as CD44^−^NK1.1^−^ stage 0 + 1, CD44^+^NK1.1^−^ stage 2, or CD44^+^NK1.1^+^ stage 3 in the thymus or as NK1.1^−^tetramer^+^ or NK1.1^+^tetramer^+^ splenocytes and liver MNCs. Numbers are % cells among total NKT cells. *n*, as in **A**. (**D**) Expression of CD4, CD69, CD122, Ly6C, Ly49C/I, and CD62L by thymic, splenic, and hepatic NKT cells was determined by flow cytometry after surface staining with specific mAb. Data are representative of 2 independent experiments. *n*, as in **A**. ns, not significant (*P* > 0.05); *P ≤ 0.05, **P ≤ 0.001, ***P ≤ 0.0001.

We previously showed that Bcl-x_L_ overexpression rescued NKT cell development in mice expressing IκB*Δ*N, a dominant-negative inhibitor of NF-κB activation^37^. Nonetheless, NKT cells that developed in these animals failed to respond to stimulation with αGalCer, a potent CD1d-restricted NKT cell agonist^14^. This lack of response to the agonist could be due to immaturity of the NKT cells that developed in *Iκ* *BΔN* ^*tg*^;*Bcl2l1* ^*tg*^ mice, their inability to process the TCR activation signals, or both. Because the genetic rescue experiment was performed in BALB/c mice, which do not express the *Klrb1c* allele that codes for NK1.1, maturation of NKT cells from NK1.1-negative to NK1.1^+^ NKT cells could not be determined^37^. Hence, we generated *Iκ* *BΔN* ^*tg*^;*Bcl2l1* ^*tg*^ mice in the C57BL/6 background (B6), which express the NK1.1-coding *Klrb1c* allele detectable with the PK136 mAb. Phenotyping of NKT cells from C57BL/6 and B6-*Iκ* *BΔN* ^*tg*^;*Bcl2l1* ^*tg*^ mice revealed that both NK1.1-negative and NK1.1^+^ NKT cells developed in B6-*Iκ* *BΔN* ^*tg*^;*Bcl2l1* ^*tg*^ mice (Fig. 1A–C). Further, the NKT cells of these mice showed phenotypic maturation as judged by expression of CD4, CD69, CD122 (IL-2/15 Rβ chain), Ly49 and Ly6C as well as the loss of CD62L expression similar to NKT cells that develop in C57BL/6 mice (Fig. 1D). Hence, both NK1.1-negative immature and NK1.1^+^ mature NKT cells escape death by thymocyte-specific Bcl-x_L_ overexpression in NF-κB signalling-deficient mice.

### PKC-θ and CARMA1-deficient mice poorly develop NKT cells

**Figure 2:**
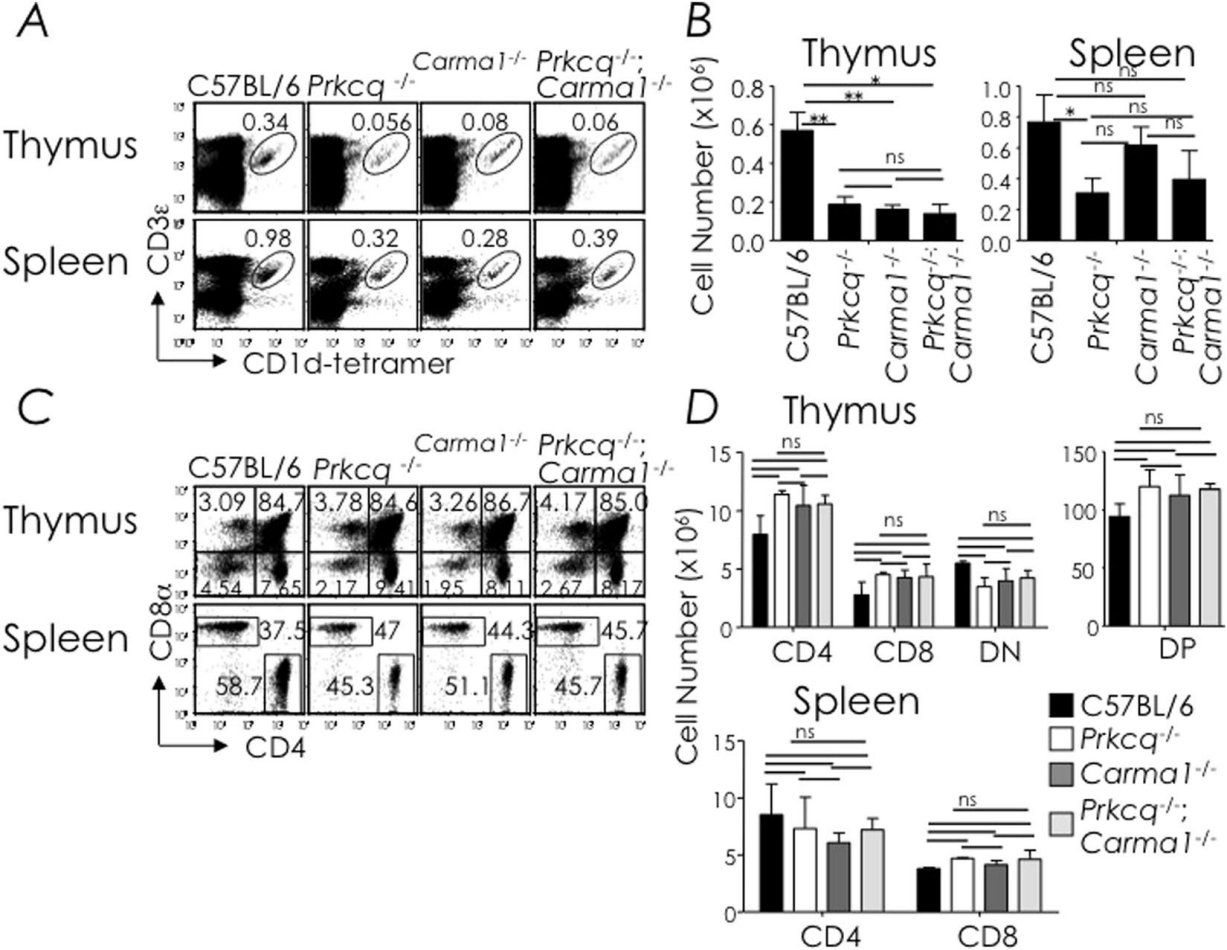
CARMA1-deficiency only partially disrupts NKT cell development. (**A**) Thymic and splenic NKT cells from C57BL/6 (*n* = 9), *Prkcq* ^−/−^ (*n* = 9), *Carma1* ^−/−^ (n = 5) and *Prkcq* ^−/−^; *Carma1* ^−/−^ (*n* = 3) mice were identified as CD3ε^+^tetramer^+^ cells within electronically gated CD8^lo^ thymocytes or B220^lo^ splenocytes. Numbers are % NKT cells among total leukocytes within each organ. (**B**) Absolute cell numbers were calculated from % NKT cell and total cell count. *n*, as in **A**. (**C**) Thymic and splenic CD4^+^ and CD8^+^ T cells from C57BL/6 (*n* = 3), *Prkcq* ^−/−^ (*n* = 3), *Carma1* ^−/−^ (*n* = 3) and *Prkcq* ^−/−^;*Carma1* ^−/−^ (*n* = 3) mice were identified as CD4^+^ or CD8^+^ cells within electronically gated thymocytes or B220^lo^ CD3ε^+^ splenocytes. Numbers are % CD4^+^ or CD8^+^ T cells among total leukocytes. Data are mean ± sem (standard error of the mean) from 3 experiments; *n*, as in **C**. (**D**) Absolute cell numbers were calculated from % CD4, CD8, CD4^+^8^+^ DP or CD4^−^8^−^ DN and total cell count. *n*, as in **C**. Data are mean ± sem from 3 (**A**,**B**) or 2 (**C**,**D**) independent experiments. ns, not significant (*P* > 0.05); *P ≤ 0.05, **P ≤ 0.001.

**Figure 3:**
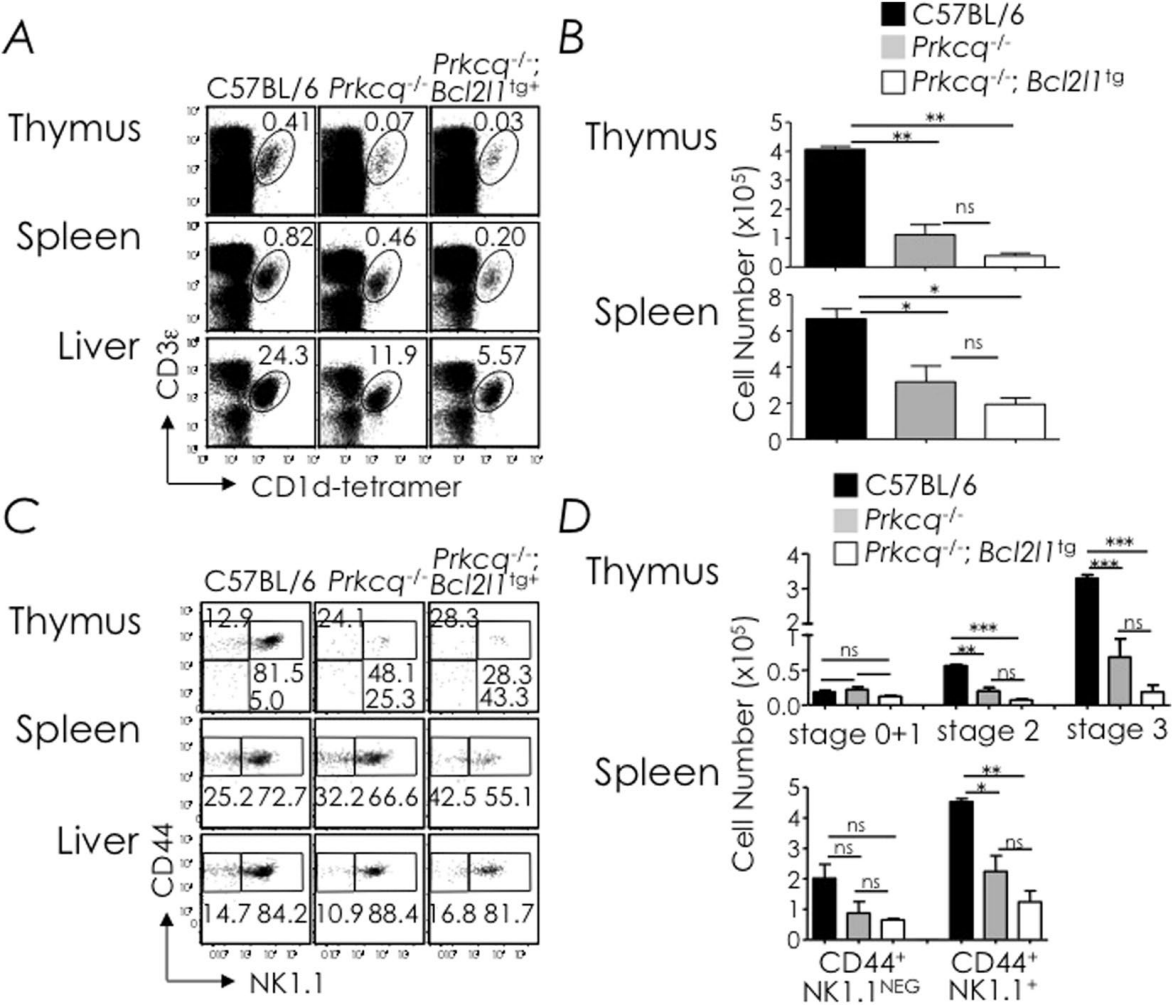
Bcl-x_L_ overexpression in PKCθ-deficient mice fails to rescue NKT cell development. (**A**) Thymic, splenic and hepatic NKT cells from C57BL/6 (n = 5), *Prkcq* ^−/−^ (*n* = 5) and *Prkcq* ^−/−^;*Bcl2l1* ^tg^ (*n* = 7) mice were identified as CD3ε^+^tetramer^+^ cells within electronically gated CD8^lo^ thymocytes or B220^lo^ splenocytes. Numbers are % NKT cells among total leukocytes within each organ. (**B**) Absolute cell numbers were calculated from % NKT and total cell count. *n*, as in **A**. (**C**) NKT cell developmental stages in the thymus, spleen and liver were identified as in Fig. 1A. Numbers are % cells among total NKT cells. *n*, as in **A**. (**D**) Absolute numbers of thymic stages 0 + 1, 2 and 3 NKT cells; they were calculated from the absolute NKT cell numbers and % stage 0 + 1, stage 2, or stage 3 cells in **C**. *n*, as in **A**. Data are mean ± sem from 3 independent experiments. ns, not significant (*P* > 0.05), *P ≤ 0.05, **P ≤ 0.001, **P ≤ 0.0001.

NF-κB activation integrates upstream signals from cell surface receptors, including both antigen receptors and death receptors. We initially focused on the antigen receptor because NKT TCRs are selected by agonistic self ligand(s). As well, we previously reported that a fraction of NK1.1-negative NKT cells that developed in *Iκ* *BΔN* ^*tg*^ mice expressed *Nr4a1*-encoded Nur77^37^. Nur77 is a TCR activation-induced pro-apoptotic transcription factor^48^, which was shown to be expressed in stage 0 precursor NKT cells^49^ and upon NKT TCR stimulation by αGalCer or bacterial glycosphingolipids^50^. Therefore, we determined whether the NKT TCR was the source of the death signal in developing NKT cells. Toward this goal, we determined the role of two NKT TCR proximal signal nodes, PKC-θ (encoded by *Prkcq*) and CARMA1. Additionally, the SLAM-SAP-FynT signalling axis partially integrates with PKC-θ^51^. As reported by us previously^14^, PKCθ-deficiency only partially impacts NKT cell development. This partial impact on NKT cell development was also reflected by CARMA1-deficiency and by combined PKC-θ and CARMA1 deficiencies (Fig. 2). Because NKT cell phenotype in PKCθ-deficient mice were reported previously (see Fig. 3 as well) and because NKT cell development in CARMA1-deficient mice reflected that of PKCθ-deficiency, NKT cell phenotype in neither type of mouse was further investigated. The results so far suggest that the membrane proximal NKT TCR-PKCθ-CARMA1 and/or SLAM-SAP-FynT-PKCθ-CARMA1 axes are unlikely required for transducing the death signal in developing NKT cells.

### Bcl-x_L_ overexpression does not rescue NKT cell development in PKCθ-deficient mice

To provide further evidence that PKCθ-CARMA1 activation does not induce a key death signal in NKT cell development, we tested whether *Bcl*-*x*_*L*_ transgene introgression into PKCθ mutant mice rescues NKT cell development. Hence, we generated *Prkcq* ^−/−^;*Bcl2l1* ^*tg*^ mice and analysed NKT cell development in them. Surprisingly, *Prkcq* ^−/−^;*Bcl2l1* ^*tg*^ mice poorly developed NKT cells akin to *Prkcq* ^−/−^ and *Carma1* ^−/−^ mice (Figs 2 and 3). Together, the data indicated that the NKT TCR and its downstream signalling modules PKC-θ and CARMA1 unlikely transduce death signals within developing NKT cells. Or alternatively, it is quite possible that Bcl-x_L_ overexpression was unable to rescue the loss of NKT cells from the extrinsic apoptotic pathway that may be induced by the NKT TCR.

### NKT cells constitutively express several TNF receptor superfamily members

**Figure 4:**
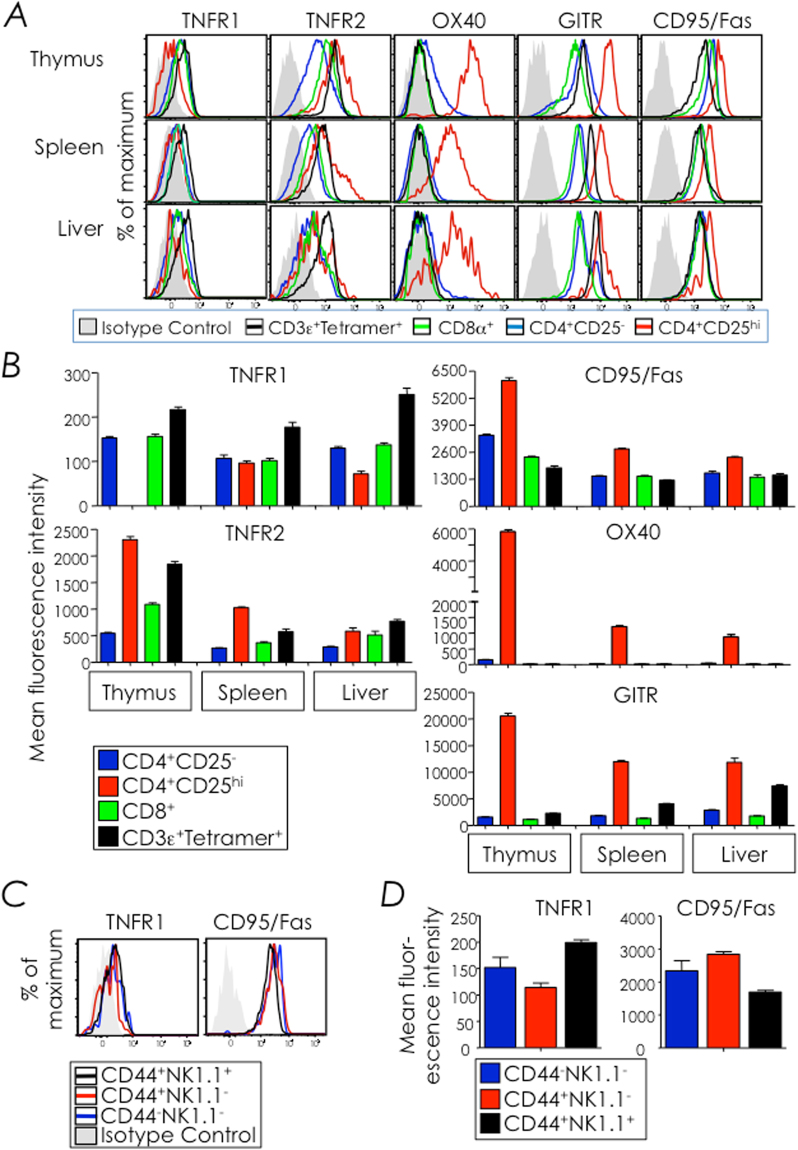
NKT cells constitutively express TNFR1 and Fas (CD95). (**A**,**B**) The expression of TNFR1, TNFR2, OX-40, GITR and Fas (CD95) by thymic, splenic, and hepatic NKT cells, CD4^+^CD25^+^, CD4^+^CD25^hi^ and CD8^+^ cells was determined by flow cytometry after surface staining with specific mAb and corresponding isotype control mAb. NKT cells from C57BL/6 (*n* = 9) mice were identified as CD3ε^+^tetramer^+^ cells within electronically gated CD8^lo^ thymocytes or B220^lo^ splenocytes. CD4^+^ and CD8^+^ T cells from mice were identified as CD4^+^ or CD8^+^ cells within electronically gated thymocytes or B220^lo^ CD3ε^+^tetramer^neg^ splenocytes and hepatic leukocytes. CD4^+^ T cells were further gated to identify CD4^+^CD25^−^ and CD4^+^CD25^hi^ cells. (**A**) Overlays show expression of the tested TNFRSF member on different T cell subsets. Filled grey histogram are representative isotype controls; note that all T cell subsets had nearly similar isotype control staining and, therefore, the staining of isotype control in CD4^+^CD25^−^ is overlaid here. (**B**) Difference in mean fluorescence intensity (ΔMFI) between specific mAb and corresponding isotype control staining on each T cell subset is plotted as mean ΔMFI ± sem; *n* and replicates as in **A**. (**C**,**D**) TNFR1 and Fas expression on thymic stage 0 + 1, stage 2 and stage 3 NKT cells was determined by flow cytometry after surface staining as in **A**. Thymic NKT cell developmental stages were identified as in Fig. 1. (**C**) Filled grey histograms are isotype controls; note that isotype control for stage 3 NKT cells are shown here as stage 1 and stage 2 NKT cells showed similar isotype control staining intensity. Representative of 3 independent experiments; *n* = 3 mice/experiment. (**D**) A plot of mean ΔMFI ± sem, computed as in **B**; *n* and replicates as in **C**.

We next turned to death domain-containing cell surface receptors as the source of death signalling in developing NKT cells. To lay focus on a particular TNFR superfamily (TNFRSF) member, we first defined TNFR1 (TNFRSF1A/CD120a), TNFR2 (TNFRSF1B/CD120b), Fas (TNFRSF6/CD95), OX40 (TNFRSF4/CD134), and GITR (TNFRSF18/CD357) expression by thymic, splenic and hepatic NKT cells. Thymic and splenic NKT cells constitutively expressed TNFR1, TNFR2, Fas, and GITR but not OX40. Of these, TNFR1 expression by NKT cells was highest amongst thymocyte subsets tested (Fig. 4A). We then focused on TNFR1 and Fas expression on the three developmental stages (stages 0 + 1, 2 and 3) of thymic NKT cells because these two TNFRSF members contain a cytoplasmic death domain. Both TNFR1 and Fas were equally expressed by all three thymic NKT cell developmental stages (Fig. 4B). Thus, NKT cells indiscriminately express the two tested TNFRSF members that contain death domains.

### Fas-deficiency fails to recue NKT cell development in NF-κB signalling-deficient mice

**Figure 5:**
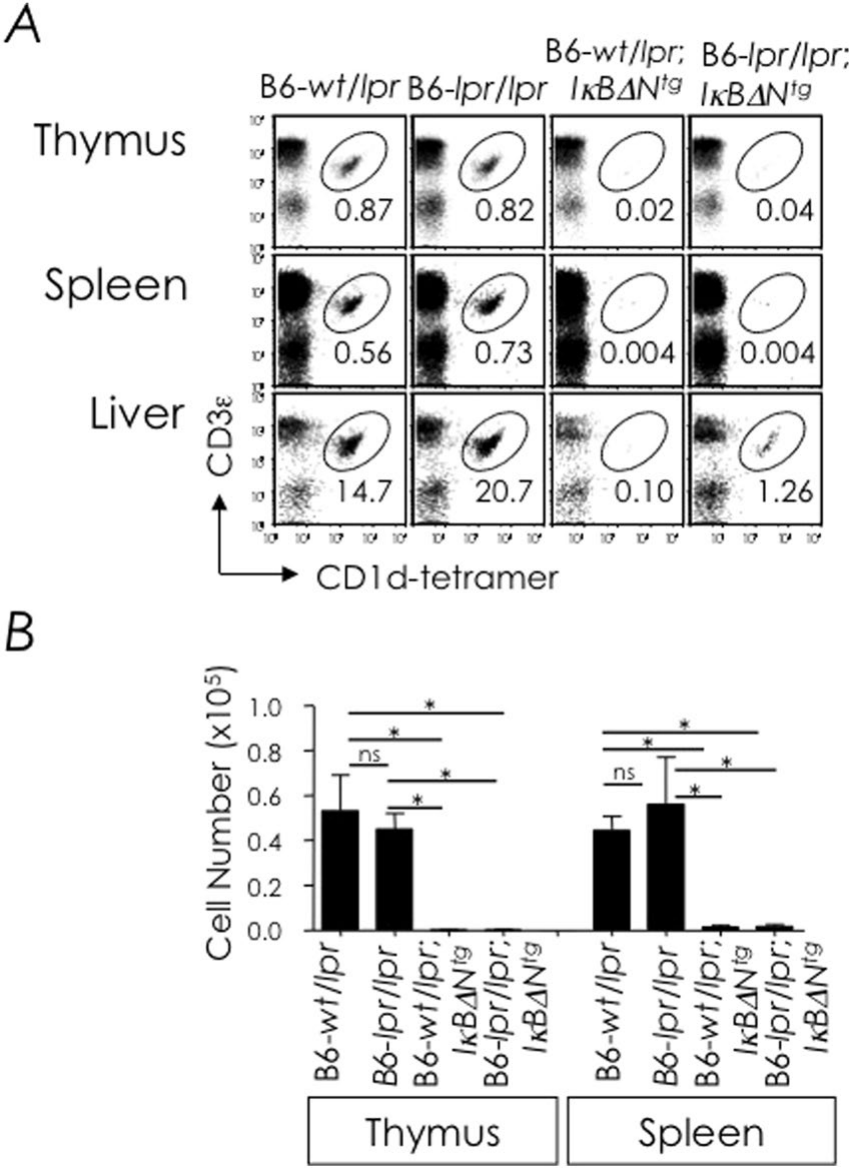
Fas-deficiency fails to rescue NKT cell development in IκBαΔN^tg^. (**A**) Thymic, splenic and hepatic NKT cells from B6-wt/lpr (n = 2), B6-*lpr*/*lpr* (*n* = 2), B6-*wt*/*lpr*;*I*κ*B*α*ΔN* ^*tg*^ (*n* = 2) and B6-*lpr*/*lpr*;*I*κ*B*α*ΔN* ^*tg*^ (*n* = 2) mice were identified as CD3ε^+^tetramer^+^ cells within electronically gated CD8^lo^ thymocytes or B220^lo^ splenocytes. Numbers are % NKT cells among total leukocytes in each organ. (**B**) Absolute cell numbers were calculated from % NKT cell and total cell count. *n*, as in **A**. Data are mean ± sem from two independent experiments. ns, not significant (*P* > 0.05), *P ≤ 0.05.

We next determined whether the expression of TNFRSF members containing a death domain altered NKT cell development. Because Fas expression was high, the development of NKT cells in B6-*lpr*/*lpr* mice, which express a nonfunctional mutant form of Fas molecule, was determined first. Thymic, splenic and hepatic NKT cells developed in the *lpr*/*lpr* mutant mice in frequencies similar to *wt*/*lpr* mice (Fig. 5). Further, the introgression of the *lpr*/*lpr* mutation into NF-κB signalling deficient *IκBΔN* ^*tg*^ mice (B6-*lpr*/*lpr*;*Iκ* *BΔN* ^*tg*^) mice did not rescue NKT cell development in these mice because very few if any NKT cells were observed in the thymus, spleen or liver of B6-*lpr*/*lpr*;*Iκ* *BΔN* ^*tg*^ mice (Fig. 5). From these data, we concluded that Fas unlikely transmits the death signal counteracted by NF-κB activation.

### Thymic NKT cells develop unhindered in TNFR1-deficient mice

**Figure 6:**
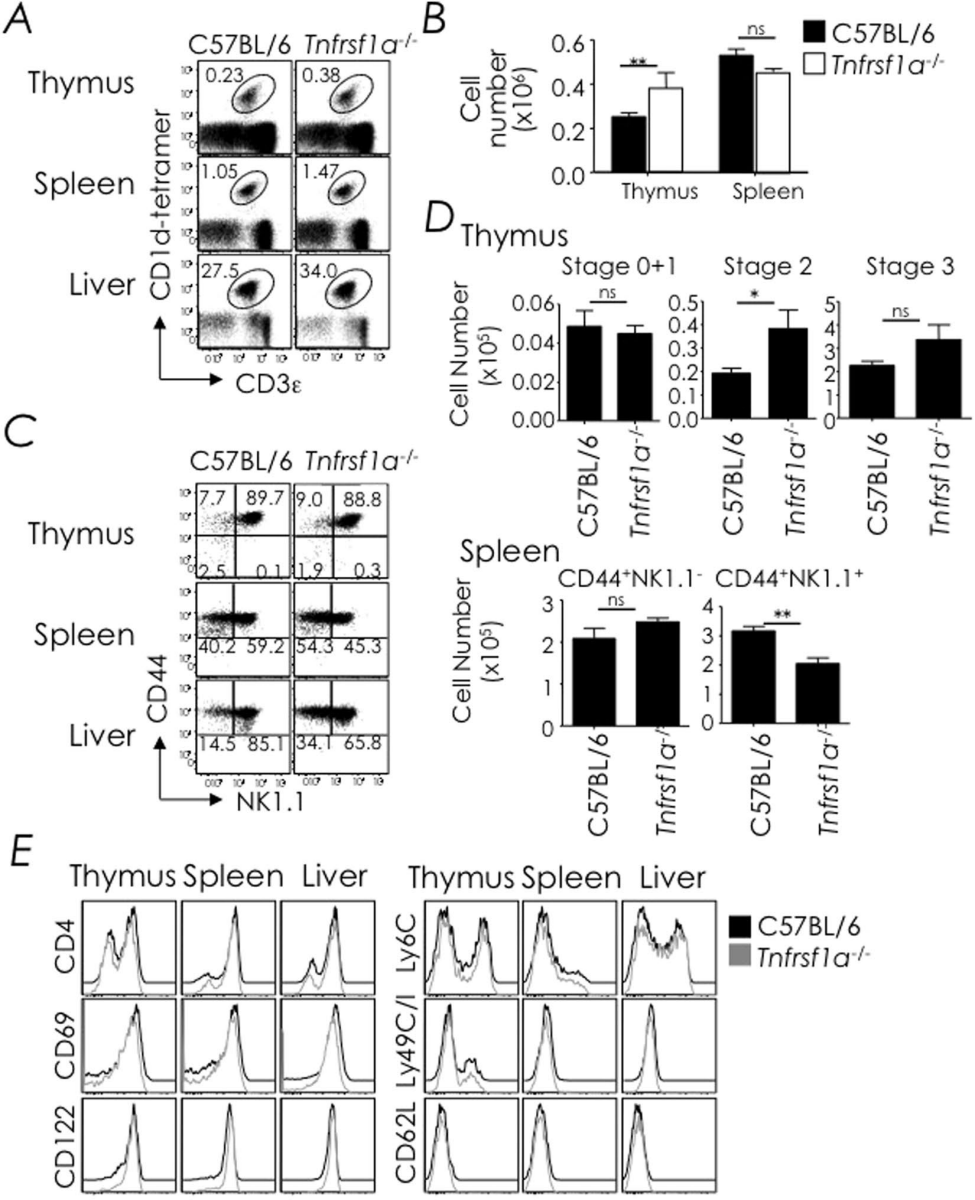
TNFR1-deficiency does not impact NKT cell development. (**A**) Thymic, splenic and hepatic NKT cells from C57BL/6 (*n* = 5) and B6.129-*Tnfrsf1a* ^−/−^ (*n* = 5) mice were identified as CD3ε^+^tetramer^+^ cells within electronically gated CD8^lo^ thymocytes or B220^lo^ splenocytes. Numbers are % NKT cells among total leukocytes within each organ. (**B**) Absolute cell numbers were calculated from % NKT and total cell count. Data are mean ± sem from 3 independent experiments; *n*, as in **A**. (**C**) Thymic NKT cell developmental stages were identified as in Fig. 1 or NKT cells were identified as splenic and hepatic NK1.1^-^tetramer^+^ or NK1.1^+^tetramer^+^ cells. Numbers are % cells among total NKT cells. *n*, as in **A**. (**D**) Absolute numbers of thymic (upper panel) stage 0 + 1, stage 2 and stage 3 NKT cells and absolute numbers of splenic (lower panel) CD44^+^NK1.1^NEG^ and CD44^+^NK1.1^+^ NKT cells; they were calculated from the absolute NKT cell numbers in **B** and % stage 0 + 1, stage 2 and stage 3 cells or CD44^+^NK1.1^NEG^ and CD44^+^NK1.1^+^ cells in **C**. *n*, as in **A**. Data are mean ± sem from 3 independent experiments. (**E**) Expression of CD4, CD69, CD122, Ly6C, Ly49C/I, and CD62L by thymic, splenic, and hepatic NKT cells was determined by flow cytometry after surface staining with specific mAb. Data are representative of 2 independent experiments. *n*, as in **A**. ns, not significant (*P* > 0.05), *P ≤ 0.05, **P ≤ 0.001.

In a similar experiment, we next determined whether TNFR1-deficiency impacted NKT cell development. The data revealed that NKT cell development proceeded unhindered in the thymus of TNFR1-deficient mice and these cells emigrated to the spleen and liver as in wild type mice (Fig. 6A,B). We noticed that the absolute NKT cell number was increased by 25–30% in the thymus, which was statistically significant, but only modestly, albeit not significantly, reduced in the spleen (Fig. 6B). Further, NKT cells proceeded to phenotypic maturity in TNFR1-deficient mice with regards to CD44 and NK1.1 expression as well as CD4, CD69, CD122 (IL-2/15 Rβ chain), Ly6C, Ly49 and CD62L (L-selectin) expression (Fig. 6C–E). Notably, the number of splenic NK1.1^+^ NKT cells was reduced in a statistically significant manner (Fig. 6D). Thus, TNFR1-deficiency marginally impacted thymic NKT cell development, whilst TNFR1 signalling was partially required for splenic NK1.1^+^ NKT cell maturation.

### NF-κB activation protects developing NKT cells from TNFR1-induced death

Deficiency in the inhibitor of κB kinase (IKK)-β, which is essential for NF-κB activation, results in poor lymphopoiesis due to enhanced sensitivity to TNF-α^52^. Consequently, normal thymopoiesis and conventional CD4 and CD8 T cell development ensues when mice are doubly deficient in TNFR1 and IKK-β^52^ suggesting that TNF-α might be a thymic inducer of NKT cell death. Therefore, we determined whether TNFR1-null mutation introgressed into *I*κ*B*α*ΔN* ^*tg*^ mice rescues NKT cells from death. For this purpose, we bred *Tnfrsf1a* ^−/−^ mice with IκBαΔN^tg^ mice and the resulting progenies were analysed for NKT cell development.

**Figure 7:**
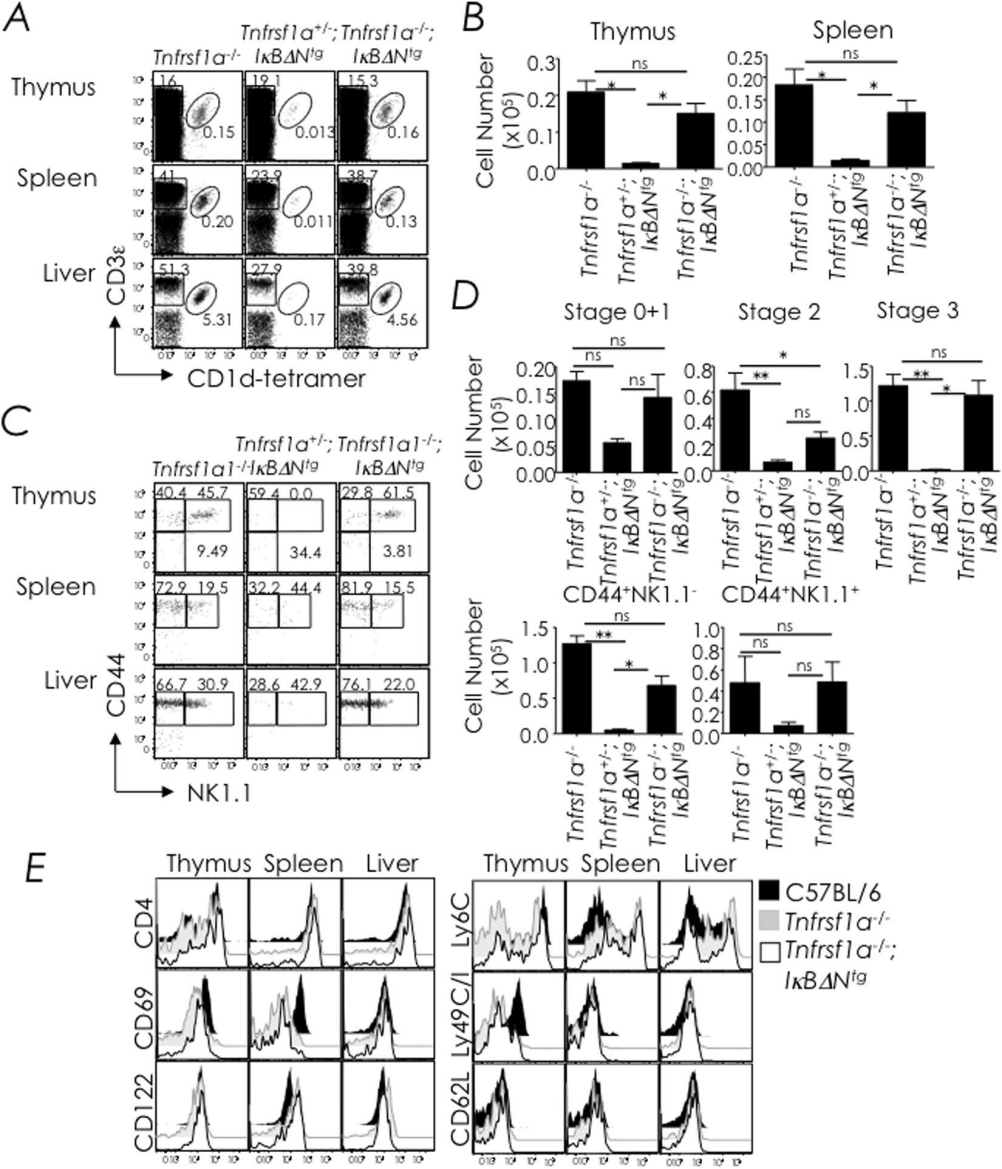
TNFR1-deficiency rescues NKT cell development in *IκB*α*ΔN* ^*tg*^ mice. (**A**) Thymic and splenic NKT cells from *Tnfrsf1a* ^−/−^ (*n* = 3), *Tnfrsf1a* ^+/−^;*IκB*α*ΔN* ^*tg*^ (*n* = 4) and *Tnfrsf1a* ^−/−^;*IκB*α*ΔN* ^*tg*^ (*n* = 15) mice were identified as CD3ε^+^tetramer^+^ cells within electronically gated CD8^lo^ thymocytes or B220^lo^ splenocytes. Numbers are % NKT cells among total leukocytes within each organ. (**B**) Absolute cell numbers were calculated from % NKT cells and total cell count. *n*, as in **A**. (**C**) Thymic NKT cell developmental stages were identified as in figure, whilst splenic and hepatic NKT cells were identified as NK1.1^−^tetramer^+^ or NK1.1^+^tetramer^+^ cells. Numbers are % cells among total NKT cells. *n*, as in **A**. (**D**) Absolute numbers of thymic stage 0 + 1, stage 2 and stage 3 NKT cells; they were calculated from the absolute NKT cell numbers and % stage 0 + 1, stage 2 and stage 3 cells in **C**. *n*, as in **A**. (**E**) Expression of CD4, CD69, CD122, Ly6C, Ly49C/I, and CD62L by thymic, splenic, and hepatic NKT cells was determined as in Fig. 1D. Data are representative of 3 independent experiments. *n*, as in **A**. ns, not significant (*P* > 0.05), *P ≤ 0.05, **P ≤ 0.001.

The data revealed that NKT cell development was rescued in *Tnfrsf1a* ^−/−^;*I*κ*B*α*ΔN* ^*tg*^ mice when compared with *I*κ*B*α*ΔN* ^*tg*^ mice (Fig. 7A,B). Even though *Tnfrsf1a* ^−/−^;*I*κ*B*α*ΔN* ^*tg*^ NKT cells matured to the CD44^hi^NK1.1^+^ stage 3 (Fig. 7C,D), they expressed lower levels of Ly49C/I (Fig. 7E) while expressing normal levels of other developmentally regulated markers (e.g., CD4, CD62L, CD122 and Ly6C) when compared to thymic *Tnfrsf1a* ^−/−^; *I*κ*B*α*ΔN* ^*tg*^ NKT cells (Fig. 7E). Because introgression of the *lpr*/*lpr* mutation into *I*κ*B*α*ΔN* ^*tg*^ mice did not rescue NKT cells from death (Fig. 5), we conclude that the effect of TNFR1-deficiency on NKT cell development in *Tnfrsf1a* ^−/−^;*I*κ*B*α*ΔN* ^*tg*^ mice is specific. The data also demonstrate that NF-κB activation controls NKT cell homeostasis by overcoming TNFR1-induced death.

### NF-κB signalling deficiency renders NKT cells sensitive to TNF-α-induced apoptosis

The data so far indicate that NF-κB signalling protects NKT cells from TNFR1-induced cell death. Further, in a previous study, we reported that NKT cells in *Iκ* *BΔN* ^*tg*^ mice, in which thymocytes are unable to activate NF-κB, expressed higher frequency of caspase 8 and caspase 9 positive cells. Forced expression of Bcl-x_L_ in *Iκ* *BΔN* ^*tg*^ NKT cells lowered the frequency of caspase 8 and caspase 9 positive cells to levels observed in wild type non-transgenic mice^37^. Guided by that finding, we designed the following experiment: C57BL/6 mouse thymocytes were cultivated in the presence of 3–24 μM BMS345541 —an inhibitor of IKK, a key node upstream of NF-κB activation^53^— or DMSO —the vehicle used to dissolve BMS345541— in the presence or absence of 10 or 100 ng/ml mouse TNF-α. After 14 hours, cells were labelled with fluorescently-tagged inhibitors for active caspase 3, caspase 8, and caspase 9. The binding of fluorescently-tagged inhibitors to active caspases allowed their detection within NKT cells by flow-cytometry. Caspase substrate tagged cells were co-stained with 7-aminoactinomycin D (7-AAD) to reveal dying/dead cells. The data presented herein are those performed with 6 μM BMS345541 treatment. This is because 12 μM BMS345541 was partially and 24 μM BMS345541 was fully toxic to thymocytes *in vitro* even in the absence of TNF-α stimulation (data not shown). Further, 3 μM BMS345541-treated thymocytes showed suboptimal caspase activation upon stimulation with 10 ng/ml and even 100 ng/ml TNF-α (data not shown).

**Figure 8:**
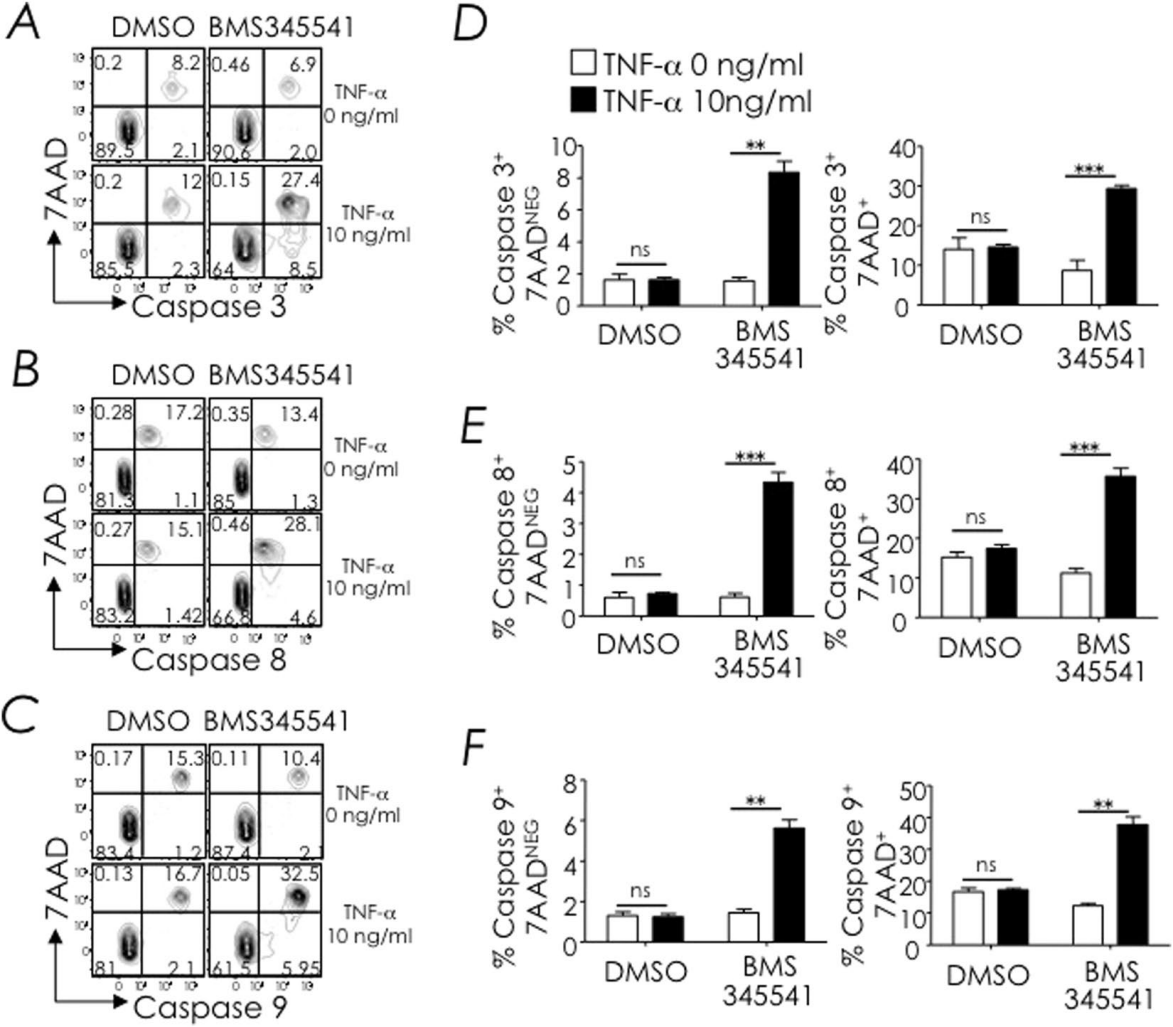
Blocking NFκB-signalling sensitises NKT cells to TNF-α-induced apoptosis. Thymocytes were treated with the IKK inhibitor BMS345541 or DMSO. After an hour, cells were washed and stimulated with indicated amount of TNF-α. After 14 hours, cells were washed and treated with fluorescently-tagged, active caspase 3 (**A**,**D**), caspase 8 (**B**,**E**) or caspase 9 (**C**,**F**) substrates separately. After four hours, NKT cells were identified by staining with anti-CD3ε and CD1d-tetramer and dying/dead cells were identified by staining with 7-AAD. Representative dot plots and quantification of caspase^+^7-AAD^+^ cells within electronically gated CD8^lo^ thymocytes are shown. Data are mean + sem from over three independent experiments. ns, not significant (*P* > 0.05), **P ≤ 0.001, ***P ≤ 0.0001.

We found that 10 ng/ml TNF-α activated caspase 3, caspase 8, and caspase 9 within thymic NKT cells in which IKK activation was inhibited with 6 μM BMS345541. In contrast, the same amount of TNF-α poorly induced cell death in NKT cells in which IKK signalling was intact (Fig. 8). This finding is consistent with the notion that NF-κB signalling is critical for protecting developing NKT cells from TNFR1-induced cell death. Furthermore, the finding that TNF-α induces both caspase 8 and caspase 9 suggested that, under the conditions tested, thymic NKT cell death is mediated by both extrinsic and intrinsic apoptotic pathways.

## Discussion

Unlike conventional T cells that are selected by antagonistic ligands^54^, several thymic T cell lineages —such as NKT cells and T regulatory cells— are selected by agonistic ligands. Agonistic ligands that positively select NKT cells in the thymus are similar or identical to the ligands that activate these cells in the periphery^55–57^. In addition to NKT TCR agonistic interactions, NKT cells persistently interact with the selecting DP cells through SLAM-SLAM interactions, which activates PKC-θ via the SAP-FynT signalling module^12,13,18,51,58–60^. Hence, we reasoned that developing NKT cells need a survival signal to protect them from death induced by high affinity NKT TCR interaction with cognate agonistic ligand and potentially SLAM-SLAM molecules as well. Consistent with this line of thinking, several laboratories^30–35,61^, including ours^37^, have found a critical requirement for NF-κB in providing a survival signal during NKT cell development. Similarly, NKT cell development partially depended on signals relayed through the TCR proximal signalling nodes, PKC-θ^14^ (this report) and CARMA1 (this report). Nonetheless, even though forced overexpression of Bcl-x_L_ partially rescued NKT development in NF-κB signalling deficient mice, the introgression of the same transgene into PKCθ-deficient mice failed to phenocopy the rescue seen in B6-*I*κ*B*α*ΔN* ^*tg*^;*Bcl2l1* ^*tg*^ mice. Hence, NF-κB provides a survival signal to NKT cells, in part through the TCR-PKCθ-CARMA1-axis, although other receptor(s) may play a role as well. This conclusion is consistent with other reports^61–64^ including the role of IL-15 in controlling NKT cell homeostasis^41–43,46,47^. Together these findings suggest that NKT cells narrowly escape death during thymic development.

The finding that TNF-α induces both caspase 8 and caspase 9 suggested that, under the conditions tested, thymic NKT cell death is mediated by both extrinsic and intrinsic apoptotic pathways. This result is consistent with our previous observation in *IκBΔN* ^*tg*^ mice wherein active caspase 8 and caspase 9 expression was increased within residual NKT cells^37^. How TNF-α induces both the intrinsic and extrinsic apoptotic pathways in NKT cells is unknown. Based on studies in hepatocytes and cancer cells, we speculate that TNFR1 ligation in NKT cells deficient in NF-κB signalling activates caspase 8 within complex II death-inducing signalling complex. Activated caspase 8 in addition to activating the executioner caspases 3 and 7 then activates caspase 9 perhaps by cleaving BH3-interacting domain death agonist (BID) to truncated BID. Truncated BID formation induces mitochondrial outer membrane permeabilization (MOMP) via B cell lymphoma 2 antagonist or killer activation. Proteins released by MOMP triggers death receptor-induced apoptosis mediated by caspase 9 apoptosome and the activation of the executioner caspases 3 and 7^65–67^. Our finding that forced BCL-x_L_ overexpression partially rescues NKT cell development in NF-κB signalling deficient *IκBΔN* ^*tg*^ mice (this report and ref.^37^) lends credence to this hypothesis.

NF-κB activation is tightly controlled in NKT cells and too little or too much signal is detrimental. Hence, the absence of RelA^31^ or inability to activate NF-κB as in the *IκBΔN* ^*tg*^ mice prevents NKT cell development^14,30^. In contrast, when the NF-κB pathway is overactive, as in mice expressing the constitutively activated IκB kinase in hematopoietic cells or mice deficient in the negative regulator of NF-κB signalling CYLD, NKT cells develop but these cells fail to mature and to populate the periphery^61^. These findings suggest that NF-κB functions as a rheostat to control the development and survival of NKT cells. In doing so, NF-κB activation perhaps sets the threshold for peripheral NKT cell activation. Setting such a threshold may be critical for agonistic ligand(s)-dependent NKT cell selection in the thymus and function in the periphery so as not to set off an autoreactive response should the agonistic ligand(s) be displayed by CD1d-positive cells in peripheral tissues such as hepatocytes and intestinal epithelial cells^68–70^. The exact mechanism by with NF-κB functions as a rheostat and sets the activation threshold remains to be elucidated.

## Materials and Methods

### Mice

Age-matched, 8–12-week old mice were used in the experiments described herein. C57BL/6, B6-*lpr*/*lpr* and B6.129-*Tnfrsf1* ^−/−^ mice were purchased from the Jackson Laboratory. B6.129-*Prkcq* ^−/−^^71^ and B6.129-*Carma1* ^−/−^^72^ were generous gifts from Drs. D.R. Littman (NYU) and V.M. Dixit (Genentech). B6-*Iκ* *BΔN* ^*tg*^;*Bcl2l1* ^*tg*^ mice were generated by crossing B6-*I*κ*BΔN* ^*tg*^ with C-*Bcl2l1* ^*tg*^, both previously described mice^14,37^, and backcrossed to the C57BL/6 background for eight generations. B6.129-*Tnfrsf1* ^−/−^;*I*κ*BΔN* ^*tg*^ mice were generated by crossing B6.129-*Tnfrsf1* ^−/−^ mice with B6-*I*κ*BΔN* ^*tg*^ mice and inter-crossing the F1 B6.129-*Tnfrsf1* ^+/−^;*I*κ*BΔN* ^*tg*^ heterozygotes to generate B6.129-*Tnfrsf1* ^−/−^;*I*κ*BΔN* ^*tg*^ homozygotes with the *Tnfrsf1* null mutation. A similar strategy was used to generate B6.129-*Prkcq* ^−/−^;*Bcl2l1* ^*tg*^ and B6-*lpr*/*lpr*;*I*κ*BΔN* ^*tg*^ mice. All mouse crosses and experiments complied with the approved protocol M/13/193 in accordance with relevant guidelines and regulations of Vanderbilt University Institutional Animal Care and Use Committee.

### Antibodies and Reagents

All antibodies (Abs) used for the identification of NKT cells and developmental stages are listed in Table 1 as described^73^. α-Galactosylceramide (αGalCer; KRN7000) was generously provided by Kirin Brewery Company (Gunma, Japan) or purchased (Funakoshi, Japan). Mouse CD1d monomer was obtained from the NIH Tetramer Facility (Emory University). Preparation of CD1d tetramers from monomers and their use are described elsewhere^73^. IKK inhibitor BMS 345541 was purchased from Sigma. Recombinant mouse TNF-α and 7AAD were purchased from BD Biosciences. CaspGLOW fluorescein Active Caspase 3 (FITC-DEVD-FMK), Caspase 8 (FITC-LETD-FMK) and Caspase 9 (FITC-LEHD-FMK) staining kit were purchased from Invitrogen.

**Table 1:** List of antibodies used for multiparametric NKT cells analyses in this study.

Antigen	Fluorochrome*	Clone	Source**
CD1d-tetramer	APC, BV421	na	in-house^73^; NIH Tetrmer Core (Emory University)
CD3ε	PE, PerCP-Cy5.5, APC, PE-Cy7	145-2C11	BD Biosciences, BioLegend, Tonbo Biosciences
CD8α	FITC, PerCP-Cy5.5, APC-Cy7	53–6.7	BD Biosciences, BioLegend, eBiosciences
CD44	FITC	IM7	BD Biosciences
NK1.1	PE, PerCP-Cy5.5, APC, APC-Cy7	PK136	BD Biosciences
B220	FITC, PerCP-Cy5.5, APC-Cy7	RA3-6B2	BD Biosciences
CD4	FITC, PE, PerCP-Cy5.5	RM4-4	BD Biosciences, BioLegend
CD69	FITC, PE, PE-Cy7	H1.2F3	BD Biosciences
CD122	PE	TM-b1	BioLegend
Ly6C	FITC, APC-Cy7	AL-21	BD Biosciences
Ly49C/I	FITC	5E6	BD Biosciences
CD62L	APC-Cy7	MEL-14	BD Biosciences
CD25	PerCP-Cy5.5, PE-Cy7	PC61	BD Biosciences
TNFR1	PE	55R-286	BioLegend
TNFR2	PE	TR75-89	BioLegend
Isotype control	PE	HTK888	BioLegend
OX40	PE	OX-86	BioLegend
Isotype control	PE	RTK2071	BioLegend
GITR	PE	DTA-1	BioLegend
Isotype control	PE	RTK4530	BioLegend
CD95/Fas	PE	Ja2	BD Biosciences
Isotype control	PE	Ha4/8	BD Biosciences

*APC, allophycocyannin; Cy, cyanine; PE, phycoerythrin; PerCP, peridinin chlorophyll protein; APC-Cy7, tandem allophycocyannin-cyanine 7 dye; **where multiple sources are indicated, the reagent worked equally well from the different sources.

### Caspase Assay

Approximately 3 × 10^6^ C57BL/6 mouse thymocytes were treated with 3–24 μM BMS345541 or DMSO control for 60 min. As 12 μM BMS345541 was partially and 24 μM BMS345541 fully toxic to thymocytes *in vitro*, and because 3 μM BMS345541 showed suboptimal caspase activation, all experiments were performed with 6 μM BMS345541 treatment. BMS345541-Treated cells were cultivated in the presence or absence of 10 ng/ml TNF-α for 14 hours. Cells were then incubated with fluorescently labelled caspase 3, caspase 8 or caspase 9 inhibitors for an additional 60 min. Cells were washed twice with wash buffer provided with CaspGLOW caspase assay kits according to the Manufacturer’s instructions. Washed cells were surface stained with CD1d tetramer and with antibodies against mouse CD3ε. Finally, cells were incubated with 7-AAD for 15 min and analyzed by FACS.

### Statistics

Comparisons between three or more groups were performed by one-way ANOVA with Tukey’s post test to determine significance. Comparison between two groups was performed by two-tailed, unpaired t test. All statistical analyses were performed using Prism (GraphPad software).

### Flow Cytometry

NKT cells were identified as CD3ε^+^tetramer^+^ cells among B220^lo^ splenocytes and hepatic mononuclear cells or CD8^lo^ thymocytes by using four-colour flow cytometry, which was performed with a FACSCalibur instrument (Becton-Dickinson). NKT cell developmental stages were phenotyped by using six-colour flow cytometry, which was performed with a LSR-II instrument (Becton-Dickinson). Data were analyzed with FlowJo software (Treestar Inc.). Absolute NKT cell numbers were calculated from % tetramer^+^ cells and total number of cells recovered from each organ.
